# Implementing Screening, Brief Intervention and Referral Intervention for Health Promotion and Disease Prevention in Hospital Settings in Alberta: A Pilot Study

**DOI:** 10.3389/ijph.2023.1605038

**Published:** 2023-02-02

**Authors:** Kamala Adhikari, Muhammad Kashif Mughal, James Whitworth, Madison Bischoff, Gary F. Teare

**Affiliations:** ^1^ Alberta Health Services, Provincial Population and Public Health, Calgary, AB, Canada; ^2^ Department of Community Health Sciences, University of Calgary, Calgary, AB, Canada

**Keywords:** health promotion, screening, feasibility, modifiable risk factors, hospital settings, brief intervention and referral (SBIR) intervention

## Abstract

**Objective:** This study assessed the feasibility of implementing screening, brief intervention and referral (SBIR) intervention in hospital settings.

**Methods:** This cross-sectional study evaluated the implementation of the SBIR intervention in a hospital in Alberta for tobacco use, alcohol intake, physical inactivity, and insufficient vegetable and fruit consumption. Patients were interviewed approximately 4-month later to collect data on the acceptability and effectiveness of the intervention received (*n* = 108). The data were primarily analyzed using descriptive statistics.

**Results:** Of 108 patients, >80% agreed that “they were ok with being screened” for the risk factors during their hospital visit. Up to 68% of patients recalled the provider’s brief education. At the follow-up, 20% of patients quit tobacco, 50% reduced alcohol use, 30% increased physical activity, and 25% increased vegetable and fruit intake.

**Conclusion:** Risk factor screening was acceptable for patients. Patients recalled the brief education they received from healthcare providers. Patients reported risk-reducing changes in their risk factors. Our future work will integrate the SBIR approach within the Electronic Clinical Information System and use robust research methods to investigate the impact of SBIR on patients’ behavior change.

## Introduction

Evidence shows that tobacco use, high alcohol intake, physical inactivity, and unhealthy diets are leading risk factors for chronic diseases and their poor outcomes for patients [1–5]. In Canada, 86% of the total cancer cases attributable to modifiable risk factors are directly related to tobacco use, excessive alcohol consumption, physical inactivity or sedentary behavior, and insufficient vegetable and fruit consumption [5–7]. In Alberta, 18% of the population smoke cigarettes, 28% engage in excessive drinking of alcohol, 70% do not get enough physical activity, and 53% do not get enough vegetable and fruit, as defined by the Canadian guidelines for a healthy lifestyle [8]. Importantly, as these risk factors are modifiable by raising awareness and encouraging behavior change, many cases of chronic diseases are preventable or manageable.

It is highly valuable to integrate health promotion and preventative interventions relating to these four risk factors into hospital care routines. These risk factors are common (43%–90%) among patients attending hospitals [9, 10], and together they account for 22% of total healthcare costs, largely driven by costs of hospital care related to diseases associated with these factors [11]. Health promotion interventions addressing these factors in hospital settings provide opportunities to support a large proportion of patients to modify their risk to prevent chronic diseases and improve treatment outcomes [12–16]. Healthcare providers are a trusted source of information about health risks, and patients who are currently experiencing ill health are more responsive to health advice for lifestyle behavior change.

Alberta Health Services (AHS) has recently developed the Health Promoting Health Services (HPHS) initiative in hospital settings in Alberta. The purpose of the HPHS initiative is to reorient Alberta’s health services to incorporate health promotion, which is one of the five strategies to promote health outlined in the Ottawa Charter for Health Promotion [17]. The framework for the initiative was developed based on the World Health Organization’s framework for Health Promoting Hospitals [18–20]. The HPHS framework facilitates the integration of health promotion into hospital programs as a core element of health service delivery, by taking actions in four domains: a) Culture of Health (engage and work together with organizational leadership and management to standardize health promotion), b) Healthier Albertans (implement health promotion activities for individual Albertans), c) Healthier Community (coordinate access to support programs and health promotion services in surrounding communities for patients and staff), and d) Healthier Site (create healthy and safe physical and social environments for patients, staff, and visitors at the site). This initiative aims to develop a suitable menu of practice change guidance and recommendations for integrating health promotion into healthcare services across Alberta.

As an initial step of the HPHS initiative, the HPHS innovation team (researchers, evaluation associates, and project managers and coordinators) focused on the Healthier Albertans domain. We initiated the implementation of screening, brief intervention and referral (SBIR) intervention in hospital settings to support patients in modifying their tobacco use, alcohol intake, physical inactivity, and insufficient vegetable and fruit consumption. The SBIR implementation used existing touchpoints between patients and providers and was directly connected to healthcare providers’ patient care mandate: healthcare providers opportunistically offered SBIR during their contact with patients for other care in hospitals.

SBIR is an integrated health promotion approach that systematically links screening, brief advice/intervention, and referral activities to timely support individuals to reduce their risk of adverse health outcomes [10, 21–25]. The SBIR approach is primarily based on the Institute of Medicine’s and World Health Organization’s recommendations for the management of alcohol dependence [21, 24, 25]. Evidence shows that SBIR is feasible to implement, is acceptable to patients and providers, and effectively reduces the risky health behaviors and improves health outcomes [10, 21–23]. However, most research has been conducted in primary care settings and emergency departments, specifically in patients with illicit drug and alcohol dependence, or with chronic diseases [21–23]. It is unclear whether the implementation of SBIR as a wider health promotion and disease prevention approach is feasible to address multiple risk factors in a variety of hospital settings [21, 23]. Our work aims to begin to address the paucity of guidance on the implementation and integration of SBIR as hospital health promotion services.

Through this SBIR implementation project, we aimed to understand the feasibility of implementing SBIR for the four modifiable risk factors in a hospital setting. This study assessed the acceptability and effectiveness of SBIR intervention in hospital settings. The primary effectiveness outcomes of the study were patients’ recall of a conversation with the healthcare provider about the risk factors that occurred during the SBIR intervention phase and their perceived improved knowledge of the link between the risk factors and chronic diseases after they received the SBIR intervention.

## Methods

### Study Design

This is a descriptive cross-sectional study design. The study used Computer Assisted Telephone Interview (CATI) to collect the data on acceptability to and effectiveness of the SBIR intervention from study participants (*n* = 108). At follow-up, CATI was conducted with the patients who received SBIR intervention, upon their consent at the time of intervention received.

### Study Setting- SBIR Implementation Setting

The HPHS innovation team engaged hospital staff in the SBIR approach between May 2019 and September 2020 in the pilot hospital, in relation to the four modifiable risk factors (tobacco use, medium or high alcohol intake, inadequate physical activity, and insufficient vegetable and fruit consumption). This is a rural hospital in the town of Alberta. Out of 40 providers in the 6 units of the pilot hospital (acute care, allied health, addictions and mental health, ambulatory chronic disease management, home care, and public health), a total of 35 providers were trained on the SBIR implementation process. Of those trained, SBIR was implemented by 24 providers (including nurses, social workers, and allied public health workers). The providers’ participation in the training/implementation was based on the human resource capacity of the unit to participate in the quality improvement work or SBIR and the clinical workload as determined by the unit managers, and the interest of the providers to participate. SBIR was initially planned to be implemented until March 2021, however implementation slowed down from March 2020 and was completely paused in September 2020 due to hospital staff reallocation to respond to the COVID-19 pandemic. This implementation project was conducted as a quality improvement project, as defined by the ARECCI (A pRoject Ethics Community Consensus Initiative) screening process for ethical project conduct.

### Study Setting- Patient Recruitment and SBIR Implementation Process

**FIGURE 1:**
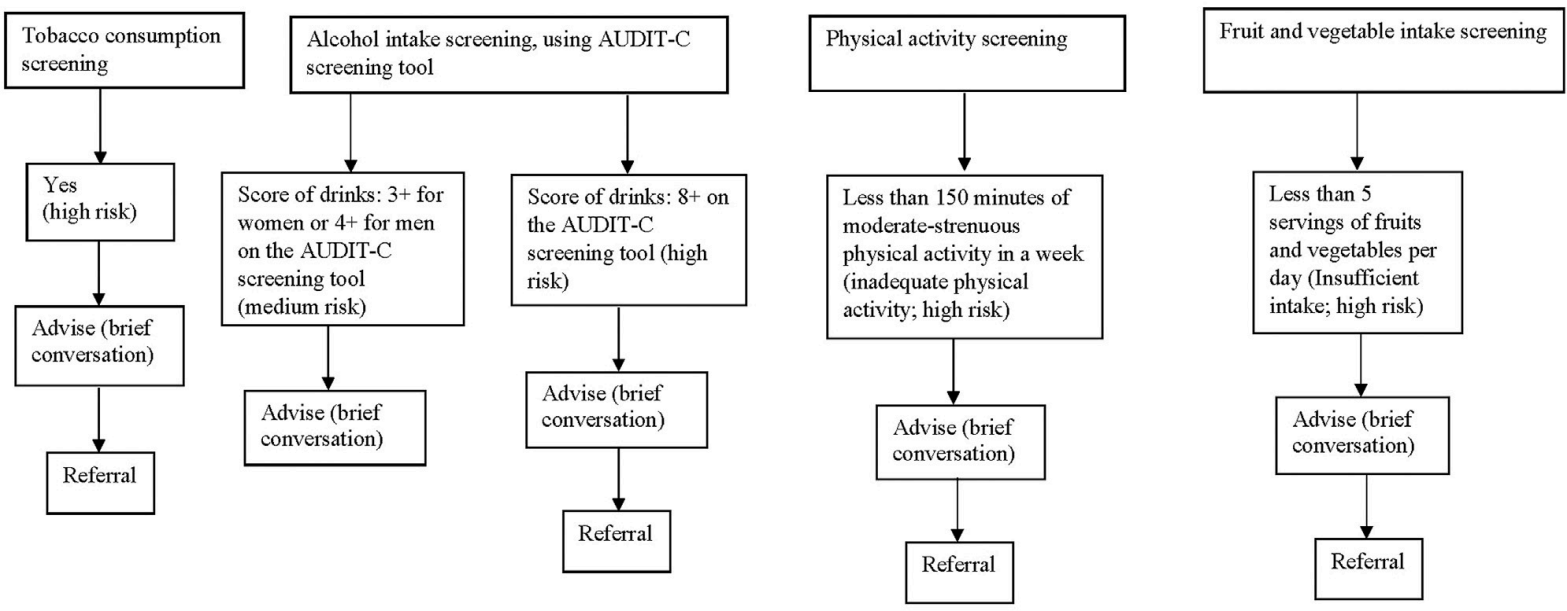
Screening, brief intervention and referral (SBIR) pathway for four modifiable risk factors, including screening questions and definitions and stratifications of increased risk groups for intervention (Alberta, Canada, 2019–2020).

The inclusion criteria for patients to be enrolled in the SBIR intervention were: a) patients aged 18 or older; b) resident of Alberta; c) cognitively and emotionally able to participate as assessed by the healthcare provider; and d) patients’ willingness to participate. Clinical providers were asked to routinely screen eligible patients for these four modifiable risk factors using standardized screening questions (*Screening*) [24, 26–31] and discuss the screening results with the patients. Patients who screened low risk on these factors were provided positive reinforcement. Patients who screened medium or high risk on these factors were offered brief, tailored advice and resources to encourage health behavior modification (*Brief Intervention*). Lastly, providers were required to offer referral(s) to health promotion programs (e.g., the AlbertaQuits smoking cessation program) in the community or within the hospital for high-risk patients (or upon patient request) who could benefit from more intensive behavior change support outside the scope of brief intervention (*Referral*). Paper-based SBIR pathways and form were designed to guide providers in the implementation process and the documentation of screening, brief intervention, referral aspects of the intervention received by each patient (Figure 1; Supplementary Material). Not all eligible patients cared for in the participating units were invited by healthcare providers to participate in the intervention. The providers were given latitude to suspend implementation periodically based on their judgement of feasibility within their clinical workflow.

### Study Setting- Implementation Strategies

The HPHS innovation team collaboratively designed the project with clinical stakeholders in the pilot hospital site (clinical providers, managers, and leaders). We engaged the clinical stakeholders throughout the SBIR intervention implementation process, including design and evaluation of the SBIR clinical workflow. The clinical stakeholders were trained on the HPHS initiative and the SBIR process. We also hired a part-time implementation facilitator who was a known nurse champion at the hospital. The facilitator supported providers throughout the implementation of SBIR through regular coaching in the use of the SBIR process and tools and integration of SBIR into their workflow. We started SBIR implementation by using a “superuser” approach, in which an individual or small group of “early adopter” healthcare providers did initial tests of SBIR implementation in their units. These super users identified barriers to the incorporation of SBIR into the unit’s workflow and worked closely with the site implementation facilitator and the innovation team to resolve issues. After the super users ironed out the SBIR workflow, over a few days to weeks, implementation was expanded to include all relevant staff stationed on that unit based on the unit’s capacity. The performance of the SBIR implementation process was regularly monitored, appraised, and feedback reports were provided (six reports over the intervention) to each participating unit and the site implementation facilitator to help maintain staff engagement and to guide improvement efforts where needed. Additionally, a member of the innovation team connected regularly, by phone, with the site providers and facilitator to discuss the SBIR implementation challenges and co-develop solutions.

### Data Collection and Key Study Variables

**FIGURE 2:**
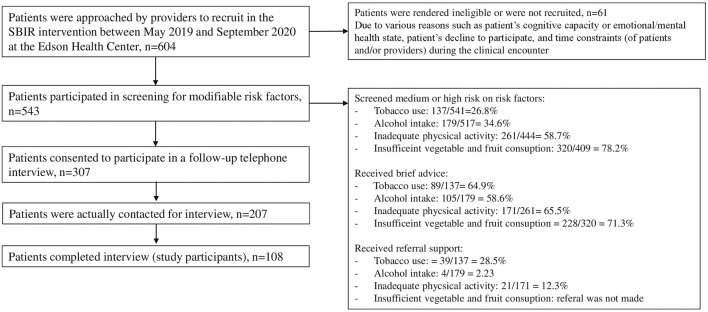
Patients receiving screening, brief intervention and referral support and their participation in follow-up interview, N = 108 (Alberta, Canada, 2019–2020).

For those patients who participated in screening for four risk factors at baseline (i.e., SBIR intervention phase), data on screening results (or risk stratification) and brief advice and referral support received status were collected using the SBIR form. At follow-up, CATI was conducted with the patients who received SBIR intervention, upon their consent, to measure the acceptability and effectiveness of SBIR. The interviews were conducted using structured questionnaires administered by the Alberta Health Services Cancer Epidemiology and Prevention Research Team, who were contracted by the HPHS innovation team to conduct the follow-up interviews. For those who did not respond to the first call, up to 6 additional calls were made, twice per day in weekdays and up to 3 messages to call back were left. Of the 543 patients screened, 307 agreed to participate and we were able to complete follow-up interviews with 108 patients (study participants, *n* = 108) (Figure 2). The median time from baseline to follow-up interview was 16 weeks (interquartile range = 12 weeks, 21 weeks). The reasons for non-participation at follow-up were: a) the follow-up calls were made during the office days/hours only, which limited the response from those who did not want to pick up the phone during that time; b) follow-up calls were put on hold during the early stages of the COVID-19 pandemic as research team members were deployed to respond to the pandemic; and c) in some cases incorrect or out-of-service phone numbers had been recorded on the study form. Additionally, a considerable number of patients did not agree to be interviewed during the calls, particularly after the start of the COVID-19 pandemic.

The study variables were [1]: patients’ acceptability of being asked, in the context of their hospital visit, about their health behaviors in relation to the risk factors, and [2] the effectiveness of the SBIR intervention in terms of (a) whether patients recalled the risk factor conversation with healthcare providers; (b) whether they perceived improvement in their knowledge of the link between the risk factors and chronic disease; and (c) patient behavior change in relation to the risk factor(s). To collect the data on acceptability, patients were asked “were you ok with being asked about whether you were a tobacco user at the hospital/clinic?” The same question was asked in relation to the other three risk factors.

SBIR effectiveness was measured by asking whether the patient (a) recalled having a conversation with the healthcare provider regarding the risk factors; (b) perceived that their knowledge of the link between the risk factors and chronic disease improved after they received the SBIR intervention. During the follow-up call, patients were also ask about their current behaviors on the risk factors and whether they had made any changes in their behavior on the risk factors since they had received the SBIR intervention in hospital. Patients’ behavior change in relation to the risk factors (i.e., reduced risk-level) in the time since they received SBIR intervention was identified based on changes between their baseline screening results during the SBIR intervention and the follow-up assessment on the risk factors. That included quitting tobacco use at least for the past 30 days, reducing the levels of alcohol use to low risk (risk score of ≤3 for women or ≤4 for men on the AUDIT-C screening tool); increasing physical activity levels to adequate levels (≥150 min of moderate-strenuous physical activity in a week); increasing vegetable and fruit consumption to sufficient levels (i.e., ≥5 servings of vegetable and fruit intake per day).

### Data Analysis

Using data from the SBIR form, we calculated the proportion of patients who screened medium or high risk on modifiable risk factors; who screened medium or high risk on modifiable risk factors, the proportion of total patients at medium or high risk who received brief advice, and the proportion of patients found to be high risk at screening who then received referral support. The sociodemographic characteristics of study participants, such as age, gender, and education status, which were collected at follow-up, were analyzed using descriptive statistics as appropriate to the data type (mean and standard deviation for continuous data; frequency and proportion for categorical data).

The proportion (and 95% confidence intervals (CI)) of the study participants who either agreed or strongly agreed that they were “ok with being asked,” during their hospital visit, about their behavior with respect to the risk factors was estimated to assess the acceptability outcome. The data collected on the various effectiveness outcomes were analyzed: the proportion (95% CI) of study participants were calculated for each effectiveness outcome. A chi-squared test was used to assess whether the proportion of participants with medium or high risk on at least one risk factor was statistically different (*α* < 0.05) between the baseline and the follow-up. Data were analyzed using SPSS version 25.0.

## Results

### Participation in SBIR Intervention and Follow-Up Interview

A total of 543 patients participated in screening for the four risk factors. The proportion of patients screened medium or high risk on the risk factors was: 27% for tobacco use, 35% for alcohol intake, 59% for inadequate physical activity, and 78% for insufficient vegetable and fruit consumption. The proportion of patients screened medium/high risk who received brief advice varied among the risk factors, ranging from 59% to 71%. Finally, the proportion of patients screened high risk who received referral support for factors ranged from 2% to 29%, varying by risk factor (Figure 2).

### Characteristics of Patients Who Participated in Follow-Up Interviews (108 Study Participants)

The mean age of the participants was 58.7 (standard deviation = 15.3). The majority of the participants were Caucasians. Approximately half of the participants had college/university level education, and most participants owned their own home. 52.8% of the participants had been initially screened at the chronic disease management unit of the hospital, with the rest screened in the other five units. Of 108 participants, 23.2% were tobacco users, 38.0% had medium/high levels of alcohol intake, 46.3% had inadequate physical activity, and 70.4% had insufficient vegetable and fruit intake at the time of screening in the hospital (Table 1).

**TABLE 1:** Characteristics of the study participants, N = 108 (Alberta, Canada, 2019–2020).

Characteristics	n (%)
Mean age	58.7 (±15.3)
Sex	
Male	55 (50.9)
Female	48 (44.4)
Missing	5 (4.6)
Ethnicity	
Caucasians	98 (90.7)
Non-Caucasian	9 (8.3)
Missing	1 (0.9)
Education	
High School or less	45 (41.7)
College/University	61 (56.5)
Prefer not to answer	1 (0.9)
Missing	1 (0.9)
Housing	
Owned with or without a mortgage	80 (74.1)
Rented	22 (20.4)
Others/Prefer not to answer	5 (4.6)
Missing	1 (0.9)
Hospital Program	
Acute Care	11 (10.2)
Allied Health	21 (19.4)
Addictions and Mental Health	4 (3.7)
Chronic Disease Management	57 (52.8)
Home Care	6 (5.6)
Public Health	9 (8.3)
Patients identified at increased risk on the modifiable factors, at initial screening^a^	
Tobacco consumption	25 (23.2)
Medium/high risk levels of alcohol intake	41 (38.0)
Inadequate physical activity	50 (46.3)
Insufficient vegetable and fruit	76 (70.4)

^a^ Sum of percentages is more than 100% as the participants were exposed to more than one risk factors.

Tobacco consumption: Any tobacco consumption in the past 30 days.

Medium/high risk level of alcohol intake: Score of drinks 3+ for women or 4+ for men on the AUDIT-C screening tool.

Inadequate physical activity: Less than 150 min of moderate-strenuous physical activity in a week.

Insufficient vegetable and fruit consumption: Less than 5 servings of vegetable and fruit per day.

### Acceptability and Effectiveness of SBIR

Of 108 participants, more than 80% participants either agreed or strongly agreed that they were ok with being screened for personal behaviors related to tobacco consumption, alcohol intake, physical activity level, and vegetable and fruits intake during their hospital visit (ranging from 80.6% for tobacco consumption to 84.3% for physical activity). At follow-up, 68.0% of participants who had been identified as tobacco users when screened at the hospital recalled having conversations with their healthcare provider related to their screening results and the modification of risk factors, which occurred at the SBIR phase. Similarly, 56.1% of participants with medium/high-risk levels of alcohol intake, 56.0% of those with inadequate physical activity, and 57.9% of those with insufficient consumption of vegetable and fruit recalled the conversations with their healthcare providers. At follow-up, of those who remembered having a conversation with their healthcare provider, the percentage of participants reporting increased understanding of the link between risk factors and chronic diseases was 82.4% for tobacco consumption, 43.5% for alcohol intake, 57.1% for physical inactivity, and 65.9% for low vegetable and fruit intake (Table 2).

**TABLE 2:** Patient-reported acceptability and effectiveness outcomes of screening, brief intervention and referral intervention in hospital settings, N = 108 (Alberta, Canada, 2019–2020).

Modifiable factors	Acceptability of SBIR^a^	Improved knowledge^b^	Reduced risk level^c^
	n/N (%) [95% CI]	n/N (%) (95% CI)	n/N (%) (95% CI)
Tobacco consumption	87/108 (80.6) [73.2, 88.2]	14/17 (82.4) [56.7, 96.2]	5/25 (20.0) [6.9, 40.8]
Alcohol intake	88/108 (81.5) [74.1, 88.7]	10/23 (43.5) [20.8,59.2]	21/41 (51.2) [35.1,67.1]
Physical activity	91/108 (84.3) [77.3, 91.2]	16/28 (57.1) [35.8, 73.7]	15/50 (30.0) [18.0, 44.7]
Vegetable and fruit intake	90/108 (83.3) [76.3, 90.4]	29/44 (65.9) [47.6, 76.9]	19/76 (25.0) [17.2, 39.1]

^a^ The number of patients either agreed or strongly agreed that they were ok with being asked, during their hospital visit, about their behavior with respect to each of the modifiable factors.

^b^ Of the patients who remembered having a conversation with their healthcare provider in hospital about the modifiable factors, the number who agreed or strongly agreed, at the time of follow-up, that their knowledge of the link between modifiable factors and the risk of chronic diseases improved.

^c^ Of the patients who screened at a medium or high risk level on the modifiable factors in hospital, the number, at the time of follow-up, who had quit tobacco use; reduced alcohol use to low-risk levels; increased physical activity levels to adequate levels; increased vegetable and fruit consumption to recommended levels.

Modifiable factors: Tobacco consumption (i.e., any tobacco use in the past 30 days), medium or high-risk levels of alcohol intake (i.e., drink score of 3+ for women or 4+ for men on the AUDIT-C screening tool); inadequate physical activity (i.e., less than 150 min of moderate-strenuous physical activity in a week); insufficient vegetable and fruit consumption (i.e., Less than 5 servings of vegetable and fruit intake per day).

Patients who screened at medium or high risk on any of the modifiable factors at the SBIR phase, and thus received the brief intervention (and referral support if high risk) reported risk-reducing changes in their behaviors. About 20.0% of those who reported tobacco consumption when screened at the hospital had stopped using tobacco at the time of follow-up. Approximately 50% of those who had medium or high risk levels of alcohol consumption at in-hospital screening reported that they had decreased alcohol consumption to low risk levels at follow-up. Similarly, 30.0% of those who were inactive or insufficiently active increased their physical activity level to a sufficiently active level, and 25.0% of those who had inadequate vegetable and fruit consumption had increased their consumption to adequate levels (Table 2). Additionally, the proportion of patients with medium or high risk on one or more of the four risk factors between the baseline (91.3%) and the follow-up (88.0%) was significantly different (*p*-value = 0.03).

## Discussion

### Key Findings and Interpretation

This study described the feasibility (mainly acceptability and effectiveness) of implementing SBIR for the four modifiable risk factors in a hospital setting. The results demonstrate the screening of four modifiable risk factors during their hospital visit is acceptable for the majority of patients interviewed at follow-up. At follow-up, the majority of patients recalled the brief education on the risk factors received from their healthcare providers and reported an improved understanding of the link between the risk factors and chronic diseases. Patients also reported risk-reducing changes in their behaviors related to the risk factors (such as quitting smoking).

SBIR in the context of a hospital visit provides an opportunity for trusted healthcare providers to educate patients about modifiable risk factors. The “brief intervention” was designed to provide educational information to patients about the risk factors. It did not include the use of motivational interviewing or further assessment of patient “readiness to change.” Many of the “referrals” were “soft referrals,” provision of information to patients on locally available support programs in their behaviour or risk factors. Furthermore, a robust referral process to support patients with excessive drinking, physical inactivity, and insufficient vegetable and fruit intake does not exist in Alberta healthcare system. Thus, we did not expect this SBIR intervention to have strong effects on patient behaviour change. Despite the more education-focused approach to brief intervention and referral—it was encouraging to see that some patients reported positive (risk-reducing) changes in behaviour on the risk factors. It is also important to note that, at the time of follow-up, most patients reported no change in their behaviors on the risk factors; some reported some levels of positive changes in their behaviors (such as reducing smoking consumption); and some reported risk increasing changes in their behaviors. It is possible that the reported changes (in both directions) at follow-up may be simply driven by measurement error or social desirability reporting bias. We recognize that robust measurement of behaviour change (effectiveness outcome) would require a more thorough approach than the self-report questions we used in a single point-in-time follow-up call without a non-intervention comparison group. Behaviour change was not our primary outcome of interest in this study.

Strong evidence exists on the effectiveness of screening and brief advice in healthcare settings on patient’s behavior change [12, 22, 23, 32, 33]. Providers’ screening and brief advice can increase the likelihood of successfully quitting [12, 22] and reduce the frequency and intensity of alcohol consumption [23, 32, 33] compared to no intervention or usual care. Additionally, various behavior support strategies, such as goal settings, action planning, feedback, informational materials, motivation, follow-up support, and pharmacotherapy, can promote the patients’ behavior change [12, 34, 35]. To our knowledge, the evidence on the effectiveness of “brief intervention” in the long-term is unclear, and the effectiveness of “referral” is yet to be studied.

### Strengths and Limitations

To our knowledge, this is the first study in Canada that assessed the acceptability and effectiveness of SBIR in hospital settings. Our SBIR intervention implementation was comprehensive, evidence-based, practical, and novel. This involved: the multiple risk factors that are common and strongly related to chronic conditions; the hospital settings that can offer influential support for positive behavior change on the significant proportion of target population at risk for (or with) chronic conditions; and the use of evidence-based strategies. Our intervention implementation was well-connected with the mission of the chronic disease prevention and management ambulatory care program at the pilot hospital. Approximately 60% of patients who participated in the SBIR intervention or at follow-up were from this unit.

This study was primarily focused on issues of implementation (feasibility and acceptability) and was less focused on ensuring strong supports were in place for patient behavior change (such as goal setting, motivational interview, and making linkages to referral resources). Not all providers in each unit performed the SBIR intervention and not all eligible patients were routinely invited to participate in the intervention. Also, for a variety of reasons, we experienced significant loss to follow-up among patients who agreed to participate in the study. The observed findings may have been biased if patients who were selected for SBIR or completed the follow-up interview were different (such as socio-demographic characteristics, clinical features, motivation for behavior change, relationship with providers) than those who were not selected or did not complete the follow-up interview (selection or attrition bias). Nevertheless, we are unable to know these differences and the influence due to data limitations. It is notable that we found a similar level of acceptability for SBIR among study participants from the chronic diseases prevention/management unit, where a higher proportion of patients were included and followed-up, as was found among the remaining units where a smaller proportion of patients participated, which suggests there may not be important selection bias in our findings with respect to that patient outcomes. Finally, self-reported data on personal behaviors known to be more prone to bias (social desirability reporting).

### Future Directions for Implementation or Health Service Practices and Research

AHS is universally implementing Connect Care (i.e., Electronic Clinical Information System) in its hospitals in Alberta. Based on the lessons learned from this paper-based SBIR implementation process and impacts, in the near future we will be leveraging Connect Care in Alberta to create and integrate a robust SBIR process (for each component “screening,” “brief intervention,” and “referral”) for the risk factors in the electronic workflows. This will enable providers to routinely and effectively support patients with risk factors. We recommend that future studies focus on investigating the impact of SBIR intervention on patients’ health behavior outcomes using a robust study design. To that end—studies should be designed to better understand the effects of educational and motivational elements of the “brief intervention” by hospital care providers and the effectiveness of linkage of patients with health behavior support services through the “referral” part of the SBIR intervention. Studies should focus on minimizing the selection bias (during patient recruitment and follow-up) and including a non-intervention comparison group. Future studies should also assess the barriers and facilitators of SBIR implementation and the effectiveness of strategies used in hospital settings, using implementation science frameworks as a guide to further improve our understanding of the feasibility of SBIR implementation. Additionally, building on the lessons learned from the SBIR implementation process and impacts, an expansion of health promotion work towards the three remaining HPHS domains is critical to direct our hospitals truly towards the health promoting hospitals or health services.

### Conclusion

Implementing a SBIR intervention focusing on multiple modifiable risk factors appears to be feasible in hospital settings. The SBIR intervention was acceptable for patients and they could recall the conversation with their healthcare provider(s) about the health risk associated with risk factors. Patients also reported improved knowledge of the link between the risk factors and chronic diseases. The lessons learned from this initial project will guide innovators, policymakers, hospital administrators, and health units in decision-making about the use or integration of SBIR process for modifiable risk factors in the future electronic workflow as routine clinical care. A robust SBIR implementation and evaluation design will be needed to further study the effectiveness of SBIR implementation in hospital settings on patients’ behavior change.

### What are the Innovations in this Policy or Program?

Our ultimate purpose is to embed health promotion as a model of care along with the disease treatment in AHS’s hospital systems. Hospital settings offer unique opportunities to support a large proportion of the population with leading modifiable risk factors of chronic conditions. We developed and integrated a paper-based SBIR process in hospital settings as routine patient care, to identify tobacco use, alcohol misuse, inadequate physical activity, and insufficient vegetable and fruit intake and support the patient’s behavior change. From this pilot, we have identified the feasibility of integrating the SBIR process in hospitals and the directions for future improvement/adaptation.

### What are the Burning Research Questions for this Innovation?

Currently, AHS is universally implementing an electronic clinical information system (the EPIC™ system, locally known as “Connect Care”) in its hospitals. Building on the initial SBIR pilot, we will develop a robust SBIR process to be integrated into Connect Care-facilitated clinical workflows. We will evaluate the feasibility of the SBIR process in Connect Care workflows and its impact on patients’ behavior change, chronic disease outcomes, and healthcare cost (using robust research methods with a non-intervention comparison group) to guide the spread, scale-up, and sustainability of the SBIR as a health promotion approach in hospitals.
