# Supplementary material for: Implementing Screening, Brief Intervention and Referral Intervention for Health Promotion and Disease Prevention in Hospital Settings in Alberta: A Pilot Study
Source: Int J Public Health. 2023 Feb 2;68:1605038. doi: 10.3389/ijph.2023.1605038 (PMC9931591; doi:10.3389/ijph.2023.1605038)
Supplement: Supplementary file 1 [file DataSheet1.PDF]

Patient label placed here (if applicable) or if labels are not used, minimum information below is required.
Name (last, first)
Birthdate (yyyy-mm-dd)
Gender
PHN#

Screening Brief Intervention Referral

Assessment/s Not Applicable for this patient ☐ Reason: ____________________

**Tobacco** ☐ Inform of AHS tobacco and smoke free environment policy	
Have you used tobacco in the past 30 days?	☐ No ☐ Yes

**Alcohol** In the past year	**0**	**1**	**2**	**3**	**4**	**Score**
How often do you have a drink containing alcohol?	Never	Monthly or less	2-4 times per month	2-3 times per week	4+ times per week	
How many standard drinks of alcohol do you drink on a typical day when you are drinking?	1 or 2	3 or 4	5 or 6	7 to 9	10 or more	
How often have you had 6 or more drinks on one occasion?	Never	Less than Monthly	Monthly	Weekly	Daily or almost daily	
					**Total Score**	
***If score of 3-7 Female or 4-7 Male with history of treatment for alcohol use -->						☐=8

**Physical Activity**	
On average, how many days per week do you participate in moderate to strenuous physical activity? (like walking fast, running, jogging, dancing, swimming, biking, or other activities that cause a light or heavy sweat)	
On average, how many minutes do you engage in physical activity at this level? (nearest 10 min)	
**Total Score**	

**Malnutrition**	
Have you lost weight in the past 6 months WITHOUT TRYING to lose weight?	☐ No to either Go to F/V
Have you been eating less than usual FOR MORE THAN A WEEK?	☐ Yes to both Go to Advice

**Fruit and Vegetable Intake**	
On a usual day, how many times do you eat fruit? Don't include the juice that you drink.	
On a usual day, how many times do you eat vegetables? Don't include French fries, fried potatoes, or chips.	
**Total Score**	

**Advice/Brochure:**	**Referral/Action:**	
☐ Tobacco Use (Positive)	☐ Tobacco Use (Positive)	--> Refer to AB Quits
☐ Alcohol Use (3+female/4+male)	☐ Tobacco Use (Positive)	--> Prescribe NRT
☐ Physical Activity (<150 min/week)	☐ Alcohol Use (8+)	--> Refer to addiction support
☐ Malnutrition (Yes to both)	☐ Physical Activity (0 min/week)	--> Refer to available programming
☐ Fruit/Veg Intake (0-4/day)	☐ Malnutrition (Yes to both)	--> Refer to dietitian
	☐ Fruit and Vegetable (<5/day)	--> Refer to available programming
	☐ Referral declined: ____________________	

**Patient Care Planning and Follow-up**	
Information associated with intervention is included in relevant patient care plan/discharge plan.	☐ Not Applicable<br>☐ Yes

To strengthen health promotion within Alberta Health Services an independent evaluator would like to contact you 1 month later to conduct a brief 10 minute phone survey.

Patient's telephone number (nnn-nnn-nnnn) REQUIRED:

Date (*yyyy-mm-dd*) **Provider Name (*printed*):** **Signature:**

Location **Department**

17539
